# Evaluation of osteopenia and osteoporosis in younger breast cancer survivors compared with cancer-free women: a prospective cohort study

**DOI:** 10.1186/s13058-018-1061-4

**Published:** 2018-11-13

**Authors:** Cody Ramin, Betty J. May, Richard B. S. Roden, Mikiaila M. Orellana, Brenna C. Hogan, Michelle S. McCullough, Dana Petry, Deborah K. Armstrong, Kala Visvanathan

**Affiliations:** 1 Department of Epidemiology, Johns Hopkins Bloomberg School of Public Health, Baltimore, MD 21205 USA; 2 Johns Hopkins Sidney Kimmel Comprehensive Cancer Center, Baltimore, MD USA; 3 The Johns Hopkins School of Medicine, Baltimore, MD USA

**Keywords:** Osteopenia, Osteoporosis, Bone loss, Breast cancer survivors, Cancer-free women

## Abstract

**Background:**

Osteoporosis, an indicator of significant bone loss, has been consistently reported among older breast cancer survivors. Data are limited on the incidence of osteopenia, an earlier indicator of bone loss, and osteoporosis in younger breast cancer survivors compared with cancer-free women.

**Methods:**

We prospectively examined bone loss in 211 breast cancer survivors (mean age at breast cancer diagnosis = 47 years) compared with 567 cancer-free women in the same cohort with familial risk for breast cancer. Multivariable-adjusted Cox proportional hazards models were used to estimate HRs and 95% CIs of osteopenia and/or osteoporosis incidence based on physician diagnosis.

**Results:**

During a mean follow-up of 5.8 years, 66% of breast cancer survivors and 53% of cancer-free women reported having a bone density examination, and 112 incident cases of osteopenia and/or osteoporosis were identified. Breast cancer survivors had a 68% higher risk of osteopenia and osteoporosis compared to cancer-free women (HR = 1.68, 95% CI = 1.12–2.50). The association was stronger among recent survivors after only 2 years of follow-up (HR = 2.74, 95% CI = 1.37–5.47). A higher risk of osteopenia and osteoporosis was also observed among survivors aged ≤ 50 years, estrogen receptor-positive tumors, and those treated with aromatase inhibitors alone or chemotherapy plus any hormone therapy relative to cancer-free women.

**Conclusions:**

Younger breast cancer survivors are at higher risk for osteopenia and osteoporosis compared to cancer-free women. Studies are needed to determine effective approaches to minimize bone loss in this population.

## Introduction

Osteopenia and osteoporosis, both systemic skeletal conditions associated with varying degrees of bone loss, are prevalent among postmenopausal breast cancer survivors, with prior reports of up to 80% experiencing loss in bone density [1]. Untreated bone loss can lead to significant morbidity due to pain and fractures, as well as to death [2]. Osteopenia is diagnosed among individuals with lower-than-average bone density, while osteoporosis is characterized by both low bone density and architectural deterioration of bone tissue [3]. Among breast cancer survivors, cancer-related risk factors for osteopenia and osteoporosis include both treatment and premature menopause [4]. Importantly, the excess risk of osteopenia and osteoporosis among breast cancer survivors, particularly those of a younger age, relative to their cancer-free peers remains unknown.

Osteopenia and osteoporosis are also prevalent in the general population. Among women aged ≥ 50 years in the United States, approximately 15.4% have osteoporosis and 51.4% have low bone density [5]. Furthermore, it is estimated that 1 in 2 women will be at risk for an osteoporosis-related fracture during their lifetime [2, 6]. Among cancer-free women, loss in bone density is associated with advancing age, menopause-induced estrogen deficiency, low body weight, lack of physical activity, excess alcohol consumption, family history of bone fracture, cigarette smoking, low calcium intake, and vitamin D deficiency [4, 7]. Loss of bone density in cancer survivors could be due to similar risk factors in addition to treatment-related effects. By comparing cancer survivors with cancer-free individuals, these risk factors can be differentiated.

Few epidemiologic studies have examined osteopenia and osteoporosis in breast cancer survivors relative to cancer-free women within the same cohort [8–10]. One prior study reported significantly lower levels of bone mineral density [8], the gold standard for assessing bone loss, and two other previous studies observed an increased risk of osteopenia and osteoporosis [9, 10] compared with cancer-free women. These studies were primarily conducted among older and long-term breast cancer survivors and did not differentiate based on tumor subtypes and detailed treatment regimens. One reason for the paucity of studies among younger breast cancer survivors is likely the challenge of identifying a comparable cancer-free group, because young cancer-free women do not routinely undergo assessment for bone health. Fortunately, we found this not to be the case in women with familial breast cancer risk, and we were therefore able to prospectively examine the risk of osteopenia and osteoporosis in a familial risk cohort known as the Breast and Ovarian Surveillance Service (BOSS) study.

## Methods

### Study population

The BOSS study is an ongoing prospective cohort study that includes women and men with familial risk for breast and/or ovarian cancer [11]. Participants were enrolled from 2005 to 2013 primarily from the Clinical Cancer Genetics Program at The Johns Hopkins Sidney Kimmel Comprehensive Cancer Center in Baltimore, MD, USA. Participants were aged ≥ 18 years with either (1) a family history of breast and/or ovarian cancer, (2) a documented *BRCA1/2* mutation, (3) a diagnosis of breast cancer at ≤ 40 years of age without a family history of breast cancer, or (4) a diagnosis of ovarian cancer at any age without a family history of ovarian cancer. Participants completed a baseline questionnaire so that information could be collected on a variety of demographic, lifestyle, and health-related factors, including detailed information on medical history and breast cancer treatment. Subsequent follow-up questionnaires have been completed every 3–4 years thereafter (> 92% have completed at least one follow-up questionnaire). Completion of the second and third follow-up questionnaires is ongoing.

For the present prospective analysis, women were included if they completed a baseline questionnaire and at least one follow-up questionnaire through September 30, 2017 (*n* = 1173). Women with a physician diagnosis of osteopenia or osteoporosis at baseline (*n* = 272 total; *n* = 174 with osteopenia only; *n* = 46 osteoporosis only) or bisphosphonate use at baseline (*n* = 5) were excluded. We further excluded women with missing responses for osteopenia or osteoporosis on baseline (*n* = 1) or follow-up (*n* = 5) questionnaires. For this analysis, breast cancer survivors were defined as women diagnosed with breast cancer (ductal carcinoma in situ [stage 0] or stages I–III breast cancer) within 5 years prior to enrollment. The comparison group was restricted to women with no prior history of cancer at baseline except nonmelanoma skin cancer or cervical carcinoma in situ. After these exclusions, 778 women (211 breast cancer survivors and 567 cancer-free) became our analytic study population.

### Exposure assessment

Cancer diagnoses were self-reported at enrollment, and pathology records were reviewed to confirm all diagnoses (by International Classification of Diseases Codes, Tenth Revision: invasive breast cancer [C50]; ductal carcinoma in situ [D05.1], and lobular carcinoma in situ [D05.0]) as well as stage and hormone receptor status (estrogen receptor [ER] /progesterone receptor [PR] and human epidermal growth factor receptor 2 [HER2]). Breast cancer treatment was reported in baseline questionnaires, and details were confirmed by medical record review (96% confirmed). Treatment information included surgery (none, lumpectomy, mastectomy) and adjuvant therapy (chemotherapy, radiation, and hormone therapy). Detailed information on type of chemotherapy and hormone therapy was also collected. We classified cancer treatment into mutually exclusive categories of surgery only, hormone therapy alone, chemotherapy alone, and chemotherapy plus hormone therapy. Hormone therapy was further classified as tamoxifen or aromatase inhibitor use.

### Outcome assessment

Osteopenia and osteoporosis diagnoses were ascertained in baseline and follow-up questionnaires. In each questionnaire, participants were asked to indicate whether they had received a physician's diagnosis of osteopenia or osteoporosis and the date of that diagnosis. Incident cases of osteopenia and osteoporosis were identified on follow-up questionnaires. Our outcome of interest was a composite outcome that included incident osteopenia (i.e., low bone mass) and/or osteoporosis. Participants also reported whether they had ever had a bone density examination and the year of examination on both baseline and follow-up questionnaires.

### Ascertainment of covariates

Information on covariates (age, race, education level, menopausal status, age at menopause, oophorectomy at a young age, body mass index [BMI], physical activity based on metabolic equivalents of task [METs] per week, alcohol intake, smoking status, hormone replacement therapy [HRT] use, current bisphosphonate use, vitamin D supplement use, and calcium supplement use) was available from the baseline questionnaire. Bilateral oophorectomy at a young age was defined as both ovaries removed prior to age 45 years and based on self-report. We calculated age at bilateral oophorectomy from the date that the second ovary was removed. Medical procedures and screening examinations, including mammograms, pap smears, sigmoidoscopy, and colonoscopy, were also reported on both baseline and follow-up questionnaires.

### Statistical analysis

Baseline characteristics of breast cancer survivors and cancer-free women were compared with frequency distributions for categorical variables and means (SDs) for continuous variables. We used Cox proportional hazards models with age as the time scale to calculate HRs and 95% CIs. Women contributed person-time from the completion date of the baseline questionnaire to the date of osteopenia or osteoporosis diagnosis or until the end of the last follow-up through September 30, 2017, whichever occurred first. The proportional hazards assumption was assessed with log-log survival plots and Schoenfeld residuals; neither method indicated that the assumption of proportional hazards was violated. Confounders were identified a priori as variables that may be associated with both breast cancer incidence and osteopenia/osteoporosis. Multivariable (MV) models were adjusted for menopausal status (premenopausal, postmenopausal), HRT use (ever, never), BMI (kg/m^2^), bilateral oophorectomy at age < 45 years (yes, no), physical activity (MET-h/wk), smoking status (ever, never), and alcohol use (g/d). To account for a small percentage of missing data (< 1% missing) in covariates, we imputed missing data with the most common category for categorical covariates and the median value for continuous covariates among cancer-free women.

To identify whether bone loss differed by subgroups of breast cancer survivors, we examined the risk of osteopenia and osteoporosis in survivors stratified by age at diagnosis, menopausal status at diagnosis, ER tumor status, and breast cancer treatment relative to cancer-free women. For models that stratified breast cancer survivors by ER status, survivors were restricted to invasive breast cancer because ER status was not routinely measured in women with a stage 0 diagnosis. We were unable to conduct analyses by HER2 status or triple-negative breast cancer, due to small numbers. We additionally conducted analyses by family history of breast cancer (no family history, first-degree relative only, first- and second-degree relatives) and an exploratory analysis by *BRCA1/2* carrier status among a subgroup of women with genetic testing.

Finally, to examine whether risk of osteopenia and osteoporosis varied by time since diagnosis, we used time since enrollment as the time metric and restricted survivors to women diagnosed with breast cancer within 1 year prior to enrollment. Models were then stratified by follow-up time (≤ 2 years and > 2 years), and heterogeneity was tested using the likelihood ratio test.

Analyses were conducted using SAS version 9.4 (SAS Institute Inc., Cary, NC, USA) and Stata version 14.0 (StataCorp LP, College Station, TX, USA) software. All statistical tests were two-sided, and *p* values ≤ 0.05 were considered statistically significant.

## Results

Age and age-adjusted baseline characteristics were compared in breast cancer survivors and cancer-free women (Table 1). Compared with cancer-free women, breast cancer survivors were more likely to be slightly older, postmenopausal, and current vitamin D users and less likely to have had a bilateral oophorectomy at a young age and a family history of breast cancer. Both survivors and cancer-free women were predominately white and highly educated (≥ 4 years of college). Among breast cancer survivors, the mean time from diagnosis to enrollment was 1.4 years, and the mean age at diagnosis was 47 years. Over 80% of breast cancer survivors were diagnosed with a first invasive breast cancer, and 76% had ER-positive breast tumors. In addition, all breast cancer survivors received surgery prior to adjuvant therapy; 65% of survivors received hormone therapy (67% tamoxifen, 41% aromatase inhibitors); and 50% of survivors received chemotherapy.

**Table 1:** Age and age-adjusted baseline characteristics of cancer-free women and breast cancer survivors in the BOSS cohort study

Characteristic	Cancer-free	Survivors^a^	*p* Value
	(*n* = 567)	(*n* = 211)	
Age^b^, years, mean (SD)	44.7 (11.3)	48.1 (10.3)	< 0.001
Race, white, %	88.7	83.3	0.02
Education, ≥ 4 years of college, %	77.6	77.4	0.72
Postmenopausal, %	27.4	51.6	< 0.001
Age at menopause^c^, years, mean (SD)	49.6 (4.9)	48.8 (3.4)	0.86
Bilateral oophorectomy at age < 45 years^d^, %	49.0	34.3	0.02
BMI, kg/m^2^, mean (SD)	26.2 (5.1)	25.9 (3.1)	0.29
Physical activity, MET-h/wk^e^, mean (SD)	29.4 (27.9)	26.0 (15.5)	0.29
Alcohol intake, g/d, mean (SD)	5.7 (7.5)	5.7 (4.8)	0.99
Smoking status, %			
Never	58.8	52.5	0.55
Former	36.7	44.8	
Current	4.2	2.7	
Missing	0.3	0.0	
HRT ever use, %	15.1	14.5	0.04
Ever mammogram^f^, %	99.5	97.8	0.63
Ever pap smear, %	98.6	99.1	0.15
Current vitamin D supplement use, %	7.8	20.8	< 0.001
Current calcium supplement use, %	25.5	28.1	0.97
Bone density examination, %	28.9	43.0	0.02
Bone density examination in women aged ≥ 45 years, %	51.2	60.0	0.08
Ever broken bone, %	6.4	6.8	0.84
Family history of breast cancer, %			
No family history	14.7	38.9	< 0.001
First-degree relative only	64.8	50.7	
First- and second-degree relatives	17.0	9.0	
Missing	3.5	1.4	
*BRCA1/2* status^g^, %			
Negative	69.7	73.9	0.33
Positive	27.3	19.4	
Variants of uncertain significance	2.9	6.7	
Age at diagnosis, years, mean (SD)	–	46.8 (10.2)	–
Time from diagnosis to baseline, years, mean (SD)	–	1.4 (1.3)	–
Invasive breast cancer (stage I–III), %	–	82.5	–
Estrogen receptor status^h^, %	–		–
Positive	–	75.9	–
Negative	–	23.6	–
Missing/untested	–	< 1.0	–
HER2 status^h^, %	–		–
Positive	–	14.4	–
Negative	–	81.6	–
Missing/untested	–	3.5	–
Triple-negative status^h^, %	–	19.0	–
Breast cancer treatment^i,j^, %	–		–
Surgery	–	100.0	–
Chemotherapy	–	49.8	–
Hormone therapy, any	–	65.4	–
Hormone therapy, by type^k^	–		–
Tamoxifen	–	67.4	–
Aromatase inhibitor	–	41.3	–

*Abbreviations: BMI* Body mass index, *HER2* Human epidermal growth factor receptor 2, *HRT* Hormone replacement therapy

Values are means (SD) or percentages and are standardized to the age distribution of the study population

^a^Women were diagnosed with stages 0–III breast cancer ≤ 5 years prior to baseline

^b^Value is not age-adjusted

^c^Among postmenopausal women

^d^Among women who had both ovaries removed (*n* = 86)

^e^Metabolic equivalents from recreational and occupational activity

^f^Among women aged ≥ 50 years

^g^Among women tested for *BRCA* status (*n* = 414)

^h^Among invasive cases only (*n* = 174)

^i^Treatment groups are not mutually exclusive

^j^Chemotherapy and hormone therapy are adjuvant

^k^Seven percent of breast cancer survivors received both tamoxifen and aromatase inhibitors (*n* = 15)

During an average of 5.8 years of follow-up, 66% of breast cancer survivors and 53% of cancer-free women reported having a bone density examination, and there were 112 incident cases of osteopenia and/or osteoporosis (75% osteopenia only). The incidence rates for osteopenia and osteoporosis were 44 cases/1000 person-years among breast cancer survivors and 19 cases/1000 person-years in their cancer-free peers. Overall, breast cancer survivors had a 68% higher risk of osteopenia and osteoporosis than cancer-free women (MV-HR = 1.68, 95% CI = 1.12–2.50) (Table 2). Results were similar when we restricted our analytic population to women who reported having a bone density examination prior to baseline and during follow-up (MV-HR = 1.90, 95% CI = 1.08–3.34; MV-HR = 1.72, 95% CI = 1.14–2.58, respectively). The results were also similar when we excluded women who had premature menopause secondary to a bilateral oophorectomy at age < 45 years (MV-HR = 1.63, 95% CI = 1.08–2.46) and only slightly attenuated when we restricted our analysis to women with no change in menopausal status during follow-up (MV-HR = 1.57, 95% CI = 0.93–2.63). Finally, the results did not change when we restricted our analytic sample to women without current vitamin D use at baseline (MV-HR = 1.68, 95% CI = 1.08–2.61) and became slightly attenuated among women without current calcium use at baseline (MV-HR = 1.59, 95% CI = 0.95–2.68).

**Table 2:** Risk of incident osteopenia and osteoporosis among breast cancer survivors compared with cancer-free women

	Events/person-years	Age-adjusted HR (95% CI)	MV-adjusted HR (95% CI)^a^
Overall			
Cancer-free	67/3509	1.00 (reference)	1.00 (reference)
Breast cancer survivors	45/1026	2.01 (1.38–2.94)	1.68 (1.12–2.50)
Excluding women without bone density examinations prior to baseline			
Cancer-free	27/1023	1.00 (reference)	1.00 (reference)
Breast cancer survivors	27/497	1.96 (1.15–3.36)	1.90 (1.08–3.34)
Excluding women without bone density examinations during follow-up			
Cancer-free	63/1890	1.00 (reference)	1.00 (reference)
Breast cancer survivors	45/703	1.89 (1.29–2.78)	1.72 (1.14–2.58)
Excluding early bilateral oophorectomy prior to baseline^b^			
Cancer-free	64/3347	1.00 (reference)	1.00 (reference)
Breast cancer survivors	42/957	1.93 (1.30–2.85)	1.63 (1.08–2.46)
Excluding pre- to postmenopausal during follow-up			
Cancer-free	34/2308	1.00 (reference)	1.00 (reference)
Breast cancer survivors	32/745	2.18 (1.34–3.55)	1.57 (0.93–2.63)
Excluding current vitamin D users^c^			
Cancer-free	60/3263	1.00 (reference)	1.00 (reference)
Breast cancer survivors	36/820	2.03 (1.34–3.08)	1.68 (1.08–2.61)
Excluding current calcium users^c^			
Cancer-free	40/2637	1.00 (reference)	1.00 (reference)
Breast cancer survivors	28/743	2.14 (1.32–3.48)	1.59 (0.95–2.68)

Abbreviations: *MV* Multivariable

^a^Adjusted for age (years), menopausal status (premenopausal, postmenopausal), bilateral oophorectomy at age < 45 years (yes, no), body mass index (kg/m^2^), physical activity (MET-h/wk), smoking status (never, ever), alcohol intake (g/d), and hormone replacement therapy (never, ever)

^b^Both ovaries removed prior to age 45 years

^c^Vitamin D and calcium supplement use was ascertained at baseline

The risk of osteopenia and osteoporosis in breast cancer survivors stratified by age at diagnosis, menopausal status at diagnosis, and ER status was compared with that of cancer-free women (Table 3). Breast cancer survivors diagnosed at age ≤ 50 years had an almost twofold increased risk of osteopenia and osteoporosis compared with cancer-free women (MV-HR = 1.98, 95% CI = 1.21–3.24). Surprisingly, the association was not significant in older women. In addition, breast cancer survivors who were premenopausal at diagnosis had increased risk of osteopenia and osteoporosis relative to their cancer-free peers (MV-HR = 1.76, 95% CI = 1.09–2.84), and this risk was similar but attenuated among women who were postmenopausal at diagnosis (MV-HR = 1.58, 95% CI = 0.86–2.89). Finally, women with ER-positive tumors had an over twofold increased risk of osteopenia and osteoporosis compared with cancer-free women (MV-HR = 2.10; 95% = 1.34–3.29). Although women with ER-negative tumors had a modest increased risk of osteopenia and osteoporosis relative to their cancer-free peers, the association was not statistically significant (MV-HR = 1.26; 95% CI = 0.54–2.94). Results were attenuated but did not differ by family history of breast cancer and *BRCA1/2* carrier status (data not shown).

**Table 3:** Risk of incident osteopenia and osteoporosis among breast cancer survivors compared with cancer-free women, stratified by characteristics at diagnosis

	Events/person-years	Age-adjusted HR (95% CI)	MV-adjusted HR^a^ (95% CI)^a^
Age at diagnosis			
Cancer-free	67/3509	1.00 (reference)	1.00 (reference)
≤ 50 years	27/651	2.34 (1.46–3.75)	1.98 (1.21–3.24)
> 50 years	18/375	1.64 (0.93–2.88)	1.34 (0.75–2.40)
Menopausal status at diagnosis			
Cancer-free	67/3509	1.00 (reference)	1.00 (reference)
Premenopausal at diagnosis	27/728	1.97 (1.24–3.12)	1.76 (1.09–2.84)
Postmenopausal at diagnosis	18/298	2.05 (1.15–3.64)	1.58 (0.86–2.89)
ER status^b^			
Cancer-free	67/3509	1.00 (reference)	1.00 (reference)
ER-negative	7/200	1.68 (0.76–3.72)	1.26 (0.54–2.94)
ER-positive	32/611	2.32 (1.52–3.55)	2.10 (1.34–3.29)

Abbreviations: *ER* Estrogen receptor, *MV* Multivariable

^a^Adjusted for age (years), menopausal status (premenopausal, postmenopausal), bilateral oophorectomy at age < 45 years (yes, no), body mass index (kg/m^2^), physical activity (MET-h/wk), smoking status (never, ever), alcohol intake (g/d), and hormone replacement therapy (never, ever)

^b^Breast cancer survivors were restricted to stages I–III

**Fig. 1:**
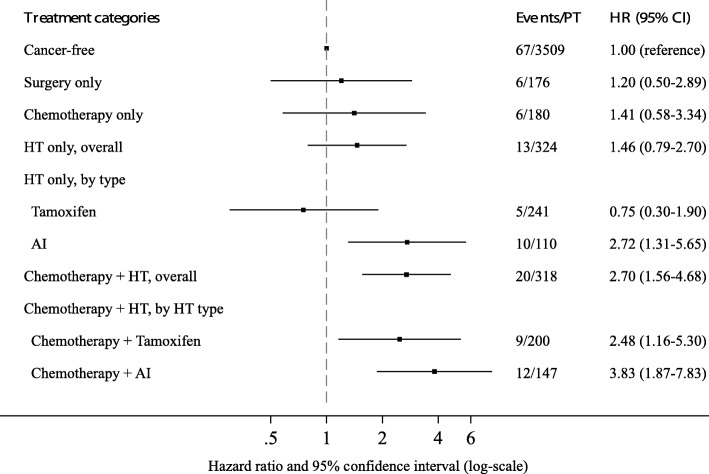
Abbreviations: Multivariable HRs (95% CIs) for incident osteopenia and osteoporosis among breast cancer survivors, stratified by type of treatment, compared with cancer-free women. Models are adjusted for Data are adjusted for age (years), menopausal status (premenopausal, postmenopausal), bilateral oophorectomy at age < 45 years (yes, no), body mass index (kg/m^2^), physical activity (MET-h/wk), smoking status (never, ever), alcohol intake (g/d), and hormone replacement therapy (never, ever). *Abbreviations: AI* Aromatase inhibitor, *HT* Hormone therapy, *PT* Person time in years

Next, the risk of osteopenia and osteoporosis in breast cancer survivors stratified by treatment compared with cancer-free women was examined (Fig. 1). Breast cancer survivors treated with chemotherapy plus hormone therapy had an over twofold increased risk of osteopenia and osteoporosis compared with cancer-free women (MV-HR = 2.70; 95% CI = 1.56–4.68). No significant association was observed for breast cancer survivors treated with surgery, chemotherapy, or hormone therapy alone compared with cancer-free women. Breast cancer survivors treated with aromatase inhibitors alone and combined chemotherapy plus aromatase inhibitors had a greater than two- and threefold increased risk of osteopenia and osteoporosis compared with cancer-free women (MV-HR = 2.72, 95% CI = 1.31–5.65; MV-HR = 3.83, 95% CI = 1.87–7.83, respectively). In addition, breast cancer survivors treated with chemotherapy plus tamoxifen had a greater than twofold increased risk compared with cancer-free women (MV-HR = 2.48, 95% CI = 1.16–5.30).

Finally, breast cancer survivors diagnosed within 1 year prior to enrollment had a greater than twofold increased risk of osteopenia and osteoporosis compared to their cancer-free peers within the first 2 years of follow-up (MV-HR = 2.74, 95% CI = 1.37–5.47) and a nonsignificant 85% increased risk of osteopenia and osteoporosis after 2 years of follow-up (MV-HR = 1.85, 95% CI = 0.98–3.51), although the *p* value for heterogeneity was not significant (*p* = 0.44) (Table 4).

**Table 4:** Risk of incident osteopenia and osteoporosis among recent breast cancer survivors compared with cancer-free women, stratified by follow-up time^a^

	Events/person-years	Age-adjusted HR (95% CI)	MV-adjusted HR (95% CI)^b^
Overall			
Cancer-free	67/3497	1.00 (reference)	1.00 (reference)
Survivors	27/475	2.49 (1.58–3.91)	2.17 (1.37–3.46)
0–2 years			
Cancer-free	22/1126	1.00 (reference)	1.00 (reference)
Survivors	14/214	3.15 (1.61–6.17)	2.74 (1.37–5.47)
3+ years			
Cancer-free	45/2371	1.00 (reference)	1.00 (reference)
Survivors	13/261	2.07 (1.11–3.85)	1.85 (0.98–3.51)

Abbreviations: *MV* Multivariable

^a^Breast cancer survivors were restricted to women diagnosed within 1 year prior to enrollment

^b^Adjusted for age (years), menopausal status (premenopausal, postmenopausal), bilateral oophorectomy at age < 45 years (yes, no), body mass index (kg/m^2^), physical activity (MET-h/wk), smoking status (never, ever), alcohol intake (g/d), and hormone replacement therapy (never, ever)

*p*(s) for the likelihood ratio test of the interaction between breast cancer status and time was 0.42 for age- and 0.44 for *MV*-adjusted models

## Discussion

To our knowledge, this is the first study to prospectively assess risk of osteopenia and osteoporosis in young and recently diagnosed breast cancer survivors compared with their cancer-free peers in a familial high-risk cohort. In this prospective study, the incidence of osteopenia and osteoporosis was almost twofold higher in breast cancer survivors than in cancer-free women over an average of 5.8 years of follow-up. The results were also similar when we excluded women with premature menopause, suggesting an effect of cancer treatment on bone health that is independent of early menopause. Breast cancer survivors who were younger, had ER-positive tumors, received aromatase inhibitors alone, or received combined chemotherapy with aromatase inhibitors or tamoxifen had a higher risk of osteopenia and osteoporosis than cancer-free women. Importantly, this was after accounting for age, menopause, and other risk factors for bone loss.

The majority of prior studies have examined bone health in breast cancer survivors without a cancer-free comparison group [12–20]. Several studies have found a higher risk of fracture in women diagnosed with breast cancer than in cancer-free women [21–23]; however, results have been inconsistent [24, 25]. Even fewer epidemiologic studies have assessed osteopenia and osteoporosis risk in women with breast cancer compared with their cancer-free peers within the same cohort [8–10]. Furthermore, these studies have included primarily older and long-term survivors. The first of these studies was conducted in the Women’s Health Initiative Observational Study (WHI-OS). This study compared the prevalence of osteoporosis and the rate of bone loss in postmenopausal breast cancer survivors compared with cancer-free women [8]. Although the investigators found that breast cancer survivors had a higher prevalence of low bone density and osteoporosis at baseline, they did not have an increased rate of bone loss compared with cancer-free women over follow-up. However, breast cancer survivors were identified from prevalent cases at study enrollment, and the time from breast cancer diagnosis to study enrollment was not reported. Therefore, it is possible that the rate of bone loss may have been assessed to late after cancer diagnosis or treatment cessation, particularly if substantial bone loss occurred shortly after diagnosis or treatment.

The second study was a retrospective registry study in the Cancer Genetics Network conducted to assess early and late effects of cancer treatment [9]. In this study, the authors assessed osteopenia and osteoporosis risk based on self-report in women with and without invasive breast cancer and found a significant positive association for both outcomes (HR = 2.1, 95% CI = 1.8–2.4 for osteopenia; HR = 1.5, 95% CI = 1.2–1.9 for osteoporosis). Although this study included younger women with familial cancer risk, breast cancer survivors were identified from 1990 to 2009, and history of bone health was collected retrospectively in 2009. Among these breast cancer survivors, over 70% were diagnosed ≥ 10 years prior to the assessment of self-reported bone health in 2009, and thus the study was susceptible to substantial recall bias.

The third study was conducted among the U.K. General Practice Research Database to examine long-term health outcomes among older cancer survivors and cancer-free individuals (overall mean age = 66.9 years, SD = 12.3 years) [10]. The authors assessed osteoporosis risk, but not osteopenia, based on medical records among breast cancer survivors and found that survivors had a 26% higher risk of osteoporosis than cancer-free women (HR = 1.26, 95% CI = 1.13–1.40). None of these studies have assessed these associations by tumor subtype or incorporated detailed information on cancer treatment and bone density examination history. In addition, only one study has previously assessed osteopenia risk [9], an earlier indication of bone loss, which is also associated with a high fracture risk.

The most common cause of bone loss in women is menopause and aging. Aging is associated with greater bone resorption and less bone formation, whereas menopause induces accelerated bone loss due to lowering levels of endogenous estrogen [26]. Therefore, a cancer-free comparison of similar age and menopausal status is important when assessing bone loss. Given that we still observed significantly higher bone loss in breast cancer survivors relative to their cancer-free peers after accounting for age and menopause, it is likely that the additional bone loss is due to the effect of treatment on bone formation.

We observed a greater than twofold increased risk of osteopenia and osteoporosis in women diagnosed with ER-positive tumors, which is likely due to hormone therapy rather than to differences in tumor biology. This is supported by the fact that the highest risk of osteopenia and osteoporosis was found among breast cancer survivors treated with aromatase inhibitors alone and chemotherapy plus aromatase inhibitors. These findings are in agreement with the underlying biology of aromatase inhibitors [27], as well as with studies in breast cancer survivors [16–20] and high-risk women in chemoprevention trials [28, 29]. Aromatase inhibitors, prescribed to postmenopausal women with ER-positive tumors, blocks the aromatase enzyme, resulting in a hypoestrogenic state associated with bone loss [27]. We found no association among women with tamoxifen use alone, a group that was primarily premenopausal at baseline (mean age at baseline = 46 years; 76% premenopausal at baseline). However, we did observe an almost twofold increased risk of osteopenia and osteoporosis among women with chemotherapy plus tamoxifen use (mean age at baseline = 43; 50% premenopausal at baseline). Although tamoxifen, a selective ER modulator, is generally thought to be protective against bone loss in postmenopausal women [30], reports suggest that it may cause bone loss among premenopausal women due to premature menopause [13, 31]. Chemotherapy may also cause bone loss due to treament-induced premature menopause in premenopausal women [32] and may have direct toxic effects on bone formation cells [27]. In addition, medications commonly presribed along with chemotherapy (e.g., corticosteroids) have also been associated with bone loss [33]. Therefore, it is biologically plausible that chemotherapy plus hormone therapy might have a joint deleterious effect on bone health early in treatment.

The strengths of this study include the prospective study design, direct comparison with cancer-free women from the same cohort, and detailed information on cancer treatment. There are also several limitations to our analysis. First, our sample size may have limited our power to detect small to moderate associations. Second, osteopenia and osteoporosis incidence was ascertained on the basis of self-reported physician diagnosis and may be susceptible to misclassification. However, 96% of women who reported a diagnosis of osteopenia or osteoporosis also reported receiving a bone density examination. Third, breast cancer survivors may have increased surveillance for bone health and therefore may be more likely than cancer-free women to be diagnosed with osteopenia and osteoporosis. In our cohort, breast cancer survivors were slightly more likely than cancer-free women to have had a bone density examination at baseline (43% vs. 29%; 60% vs. 51% among women aged ≥ 45 years) and in follow-up (66% vs. 53%). However, sensitivity analyses to further reduce the possibility of undetected prevalent or incident cases found that results were similar when restricted to women with bone density examinations prior to baseline (MV-HR = 1.90, 95% CI = 1.08–3.34) and during follow-up (MV-HR = 1.72, 95% CI = 1.14–2.58). Furthermore, both women with and without breast cancer in our cohort underwent close monitoring for their health. Specifically, overall health screening history at baseline was similar in breast cancer survivors compared with cancer-free women (e.g., 99% vs. 99% had ever had a pap smear; 100% vs. 98% had ever had a mammogram among women aged ≥ 50 years). Finally, our results may not be generalizable to other populations, because our study population was composed predominately of white and highly educated women at high risk for breast cancer. However, we believe that the underlying biology of cancer treatment and its effect on bone health are likely similar across ethnicities. The homogeneity of our study population also improves the internal validity of this study because it reduces the influence of potential unmeasured factors.

## Conclusions

In summary, our results demonstrate that incident osteopenia and osteoporosis are significantly higher in young breast cancer survivors within a few years of diagnosis than in cancer-free women and that risk varies by cancer treatment. These findings provide support for a baseline evaluation of bone density and fracture risk assessment close to breast cancer diagnosis, particularly among young survivors being treated with combined chemotherapy and hormone therapy, so that prevention strategies and appropriate monitoring can be implemented early. Future studies are needed to address the frequency of monitoring in breast cancer survivors by specific age and treatment groups.

## Abbreviations

BMI: Body mass index
BOSS: Breast and Ovarian Surveillance Service
ER: Estrogen receptor
HER2: Human epidural growth factor receptor 2
HRT: Hormone replacement therapy
METs: Metabolic equivalents of tasks
